# Microfluidization-Driven Structural Reorganization and Functional Improvements of Whole Chickpea Flour

**DOI:** 10.3390/foods15132293

**Published:** 2026-06-26

**Authors:** Jonathan Chen, Harshi Singhi, Yaren Yurdagul, Oguz Kaan Ozturk

**Affiliations:** Department of Food Science and Human Nutrition, University of Illinois at Urbana-Champaign, Urbana, IL 61801, USA; exc2@illinois.edu (J.C.); hsinghi2@illinois.edu (H.S.); yareny2@illinois.edu (Y.Y.)

**Keywords:** chickpea flour, functionality, microfluidization, plant-based

## Abstract

The increasing global demand for dietary protein has intensified the search for functional and sustainable plant-based ingredients. Chickpea flour is a promising candidate owing to its high nutritional quality and rich bioactive content. This study evaluated the use of microfluidization as a non-thermal strategy to enhance the physicochemical and functional properties of chickpea flour. Microfluidization induced particle fragmentation and led to protein denaturation, producing more irregular and porous surface morphologies. These structural modifications increased surface hydrophobicity, enhancing emulsifying and foaming capacities. Enhanced surface hydrophobicity also led to marked improvements in oil-holding capacity (up to 210% increase over control, after microfluidization at 200 MPa for three passes), likely due to stronger interactions with non-polar solvents. In parallel, microfluidization facilitated greater protein-water interactions, resulting in a 210% increase in protein solubility and 40% improvement in water-holding capacity after microfluidization at 200 MPa for one pass, compared to control. Increased surface area additionally contributed to higher in vitro protein digestibility (about 45% higher than control for all microfluidized samples) and the formation of a stronger network. Overall, these results demonstrate that microfluidization is an effective approach for improving the functional performance of whole chickpea flour, supporting its potential application in plant-based food systems.

## 1. Introduction

Rapid global population growth, coupled with shifts in dietary preferences toward higher-protein diets, has substantially increased worldwide consumption of dietary proteins [1]. Although animal-derived proteins possess a holistic amino acid profile and generally exhibit superior functional properties, plant-based proteins are increasingly favored due to environmental sustainability concerns, ethical considerations, and economic constraints associated with animal protein production [2,3,4,5]. Currently, wheat gluten, soy protein, and pea protein dominate the plant protein market. However, allergenicity issues, supply limitations, and lack of diversity among commercially available protein sources highlight the need to identify underutilized plant proteins to meet growing global nutritional and functional demands [6].

Chickpea (*Cicer arietinum*) is a widely consumed pulse recognized for its rich nutritional profile and abundance of bioactive compounds [7]. Chickpea protein is considered to be of relatively high quality among legumes, with a balanced amino acid composition and good digestibility [8]. Chickpea flour has a relatively high protein content (18–28%) and substantial proportion of amylose, which plays a significant role in its gelatinization behavior [9]. As in other pulse flours, the functional performance of chickpea flour is governed not only by its protein and starch contents but also by interactions among these components, which can be significantly altered by processing conditions. Accordingly, recent studies have explored the effects of processing technologies such as high-pressure homogenization [10] and extrusion [11] on the physicochemical and functional properties of chickpea flour. It has also been directly incorporated into food systems, particularly extruded products [12,13]. Yet, despite these advances, the potential of microfluidization as a targeted approach to improve functional performance of chickpea flour remains largely unexplored.

Microfluidization has emerged as a promising high-shear, non-thermal processing technology for modifying the structural and functional properties of plant-based ingredients [14]. The process involves forcing fluids through microchannels at elevated pressures, resulting in intense shear, collision, and cavitation forces that lead to substantial particle size reduction and improved particle uniformity [15]. Unlike other non-thermal technologies, such as ultrasonication [16] and cold atmospheric plasma [17], microfluidization does not produce significant amounts of free radicals or reactive species, thereby minimizing nutrient degradation and preserving natural antioxidants. Compared with conventional high-pressure homogenization, microfluidization produces particles that are smaller and more homogenous. This is due to a higher and constant shear rate, achieved through the use of fixed geometry microchannels instead of valves, as well as utilizing an intensifier pump [18].

During microfluidization, materials are exposed to intense pressure and shear forces, followed by a rapid pressure drop, inducing cavitation [19] and effectively disrupting cellular and macromolecular structures. These effects can induce protein unfolding, aggregation, rearrangement, and enhanced protein-polysaccharide interactions, ultimately improving solubility, emulsifying, foaming, and gelling properties [20]. Microfluidization has therefore been successfully applied to improve the functionality of plant-based ingredients, including whole cowpea flour, where increases in the water- and oil-holding capacities were reported [21].

Based on these considerations, we hypothesized that microfluidization would enhance the functional performance of chickpea flour by modifying protein and polysaccharide structures, improving protein solubility, emulsifying properties, and hydration characteristics. Hence, the main objective of this study was to assess the effects of microfluidization pressure (100 and 200 MPa) and number of passes (1 and 3 passes) on the physicochemical, structural, and functional properties of chickpea flour. This work aims to evaluate microfluidization as a sustainable and scalable processing strategy for improving the techno-functional performance of whole pulse flours for plant-based food applications.

## 2. Materials and Methods

### 2.1. Materials

Chickpea flour (CPF) was obtained from ADM (Decatur, IL, USA). Canola oil was purchased from a local retailer (Walmart, Champaign, IL, USA). Hexanes (≥98.5%), Tris (≥99.8%), glycine (≥98.5%), methanol (≥99%), glacial acetic acid (≥99.9%), bovine serum albumin (BSA) standard, and Bradford reagent were obtained from Thermo Fisher Scientific Inc. (Waltham, MA, USA). 5,5′-dithiobis-(2-nitrobenzoic acid) (DTNB) (≥98%) and 8-Anilinonaphthalene-1-sulfonic acid (ANS) (≥97%) were obtained from Sigma-Aldrich (St Louis, MO, USA). All other reagents were of analytical grade and obtained from Thermo Fisher Scientific Inc. (Waltham, MA, USA).

### 2.2. Proximate Analysis of CPF

The moisture content of the CPF was determined by drying at 105 °C for 24 h until a constant weight was achieved, according to ISO 771:2021 [22]. Nitrogen content was measured following the Dumas method [23] using a combustion analyzer (LECO FP828, LECO Corporation, St Joseph, MI, USA) and protein content was calculated using a conversion factor of 6.25 [24]. The ash content was determined by combustion of the samples at 550 °C for 24 h in a muffle furnace (Isotemp 550, Thermo Fisher Scientific Inc.) according to AACC method 08-03.01 [25]. The total lipid content was quantified using the Soxhlet extraction method with hexanes according to ISO 659:2009 [26]. Carbohydrate content was calculated as the remaining mass after subtracting all the other components measured.

### 2.3. Microfluidization of CPF

CPF (5%, *w*/*v*) was stirred in deionized water for three hours to produce CPF dispersions. The prepared dispersions were subjected to microfluidization using a benchtop microfluidizer (M-110P, Microfluidics, Westwood, MA, USA). Samples were processed at either 100 or 200 MPa for one or three passes, using an 87 µm Z-type interaction chamber. Treatments were designated as 100 MPa–1 (100 MPa for one pass), 100 MPa–3 (100 MPa for three passes), 200 MPa–1 (200 MPa for one pass), and 200 MPa–3 (200 MPa for three passes). During microfluidization, the interaction chamber and cooling coil were immersed in an ice bath to minimize any thermal effects. A non-microfluidized CPF dispersion (0 MPa), also stirred for three hours at room temperature, served as the control. Following treatment, aliquots were lyophilized for solid-state analyses, while the remaining dispersions were used for aqueous-state measurements.

### 2.4. Physicochemical Characterization

#### 2.4.1. Particle Size

The particle size distributions were measured with a SALD-2300 laser diffraction particle size analyzer (Shimadzu, Kyoto, Japan), following dilution to an absorbance reading of approximately 0.100 with deionized water. Each reading was taken after the absorbance reading was stable for a minimum of one minute to ensure a homogenous sample. The median particle diameter (D50), calculated using a logarithmic Gaussian distribution function, was used for subsequent principal component analysis (PCA).

#### 2.4.2. Zeta Potential

CPF dispersions were diluted 25-fold with 0.01 M potassium phosphate buffer (pH 7.0). Zeta potential was measured at 25 °C using a Brookfield NanoBrook Omni analyzer (Brookhaven Instruments Corporation, Holtsville, NY, USA) operating in phase analysis light scattering (PALS) mode, with calculations based on Smoluchowski model.

#### 2.4.3. Free Sulfhydryl Groups

The free sulfhydryl group content was determined based on the reaction between DTNB and free sulfhydryl groups [27] as described by Chen & Ozturk [28]. The samples were diluted to 1 mg protein/mL in 0.01 M potassium phosphate buffer (pH 7.0). DTNB solution (60 µL,10 mM in 0.1 M Tris-HCl buffer) was added to 1.8 mL aliquots of diluted samples, followed by incubation at 40 °C for 15 min. The mixtures were filtered (0.20 µm), cooled to 25 °C, and absorbance was measured at 412 nm using a BioTek Microplate Reader (Agilent Technologies, Santa Clara, CA, USA). Blank samples, made by adding 0.1 M Tris-HCl buffer instead of DTNB solution, were also recorded. The free sulfhydryl content was determined using the following equation.

(1) $$Free\;Sulfhydryl\;Groups\;(\mu mol/g)=\frac{\left(A_{s}-A_{b}\right)\times 73.53}{C}$$

where *A_s_* and *A_b_* represent sample and blank absorbance, respectively, and *C* is the protein concentration (1 mg/mL). 73.53 represents the conversion from M to µM and divided by 1.36 × 10^4^ M^−1^ cm^−1^, the molar extinction coefficient of 2-nitro-5-thiobenzoate ion (TNB^2−^).

#### 2.4.4. Surface Hydrophobicity

Surface hydrophobicity was analyzed using ANS as a fluorescent probe, based on the procedure described by Chen & Ozturk [28] and previously verified by Alizadeh-Pasdar & Li-Chan [29]. CPF dispersions were diluted to obtain protein concentrations of 0.05, 0.1, 0.2, and 0.4 mg/mL. For each concentration, 1 mL of the diluted sample was mixed with 5 µL of the ANS solution (8 mM in 0.1 M Tris buffer, pH 8.0), briefly vortexed, and incubated at 25 °C for 10 min to facilitate probe binding. The samples were centrifuged (7745× *g* for 10 min) and 250 µL of the resulting supernatant was collected and loaded into black 96-well microplates. Blank wells filled with 0.1 M Tris buffer (pH 8.0) served as the negative control during fluorescence analysis. Fluorescence was recorded using a SpectraMax M5 Microplate Reader (Molecular Devices, San Jose, CA, USA) at 390 nm excitation and 470 nm emission. Surface hydrophobicity was represented by the slope of the corrected fluorescence intensity–protein concentration curve.

### 2.5. Functional Properties of Microfluidized CPF

#### 2.5.1. Protein Solubility

The Bradford assay was used to quantify protein content [30]. A standard curve was prepared using BSA within the linear range of 0.031–2 mg/mL. Following centrifugation (7745× *g* for 10 min), 20 µL of supernatant was reacted with 1000 µL of Bradford reagent and gently inverted. The mixtures were transferred to a 96-well microplate and incubated for 10 min at room temperature. Absorbance was recorded at 595 nm using a BioTek Microplate Reader (Agilent Technologies, Santa Clara, CA, USA) against a reagent blank for baseline correction.

#### 2.5.2. Emulsifying Properties

The emulsifying activity index (EAI) and emulsion stability index (ESI) were measured according to the methodology developed by Pearce & Kinsella [31], with modifications as described by Chen & Ozturk [28]. CPF dispersions, diluted to 1% (*w*/*v*) with deionized water, were mixed with canola oil (3:1, *v*/*v*) and homogenized with a T25 Ultra-Turrax homogenizer (IKA, Staufen, Germany) at 15,000 rpm for 2 min. Aliquots (100 µL) of the emulsions were immediately diluted into 3 mL of 0.1% SDS solution. The absorbance at 500 nm was recorded using a GENESYS™ 30 UV-Vis spectrophotometer (Thermo Fisher Scientific Inc., Waltham, MA, USA). After 30 min, another aliquot was similarly diluted and absorbance at the same wavelength was measured to assess emulsion stability. EAI and ESI were calculated using the following equations.

(2) $$EAI\left(m^{2}/g\right)=\frac{2\times 2.303\times A_{0}\times DF}{C\times \varphi \times \theta \times 1000}$$

(3) $$ESI\left(min\right)=\frac{A_{0}}{A_{0}-A_{30}}\times 30$$

where *A*_0_ is the absorbance measured immediately following homogenization (t = 0 min), *DF* represents the dilution factor (30), *C* is the protein concentration (10 mg/mL), *φ* represents the path length (0.01 m), *θ* is the ratio of oil volume to total volume (0.25), and *A*_30_ is the absorbance measured after standing for 30 min (t = 30 min).

#### 2.5.3. Foaming Properties

Foaming capacity (FC) and foaming stability (FS) were evaluated following the procedure described by Chen & Ozturk [28]. A 15 mL portion of the CPF dispersion was transferred into a measuring cylinder and aerated using a T25 Ultra-Turrax homogenizer (IKA, Staufen, Germany) at 13,400 rpm for 2 min at 25 °C. The foam surface was carefully leveled using a spatula and the foam volume was recorded at 0 and 30 min. FC and FS were calculated using the following equations.

(4) $$FC\left(\%\right)=\frac{V_{0}-V}{V}\times 100\%$$

(5) $$FS\left(\%\right)=\frac{V_{30}-V}{V_{0}-V}\times 100\%$$

where *V*_0_ is the total volume immediately following homogenization (t = 0 min), *V* is the initial volume of sample (15 mL), and *V*_30_ is the total volume after 30 min (t = 30 min).

#### 2.5.4. Water- and Oil-Holding Capacities

Water- and oil-holding capacities (WHC and OHC) were determined following the method described by Singhi & Ozturk [32], with slight modifications. For WHC, lyophilized CPFs (0.5 g) were mixed with deionized water (10 g) in a pre-weighed centrifuge tube and thoroughly dispersed by continuous stirring at 60 °C for 40 min to ensure complete hydration. The mixtures were then cooled to room temperature and centrifuged (7745× *g* for 10 min) to separate unbound water. The supernatant was decanted, and the tube was weighed again. WHC was calculated using the following equation.

(6) $$WHC\left(\%\right)=\frac{m_{1}-m_{0}}{m_{0}}\times 100\%$$

where $m_{0}$ is the mass of the dry CPF sample, and $m_{1}$ is the mass of the sample after water absorption.

For OHC, lyophilized CPFs (0.5 g) was thoroughly mixed with canola oil (5 g) to ensure uniform dispersion. The mixtures were kept at room temperature for 30 min to facilitate oil absorption. The mixtures were centrifuged (6365× *g* for 20 min) to separate unbound oil. OHC was calculated using the following equation.

(7) $$OHC\left(\%\right)=\frac{m_{2}-m_{0}}{m_{0}}\times 100\%$$

where $m_{0}$ is the mass of the dry CPF sample, and $m_{2}$ is the mass of the sample after oil absorption.

### 2.6. Molecular Characterization

#### 2.6.1. Fourier-Transform Infrared Spectroscopy

Fourier-transform infrared (FTIR) spectroscopy was conducted in attenuated total reflectance (ATR)-FTIR mode, using a Spectrum Two FTIR spectrometer (PerkinElmer, Waltham, MA, USA), to characterize the structural properties of the CPF samples. Lyophilized CPF powder samples were directly placed onto the ATR crystal and scanned over the wavenumber range of 4000–550 cm^−1^ with 64 scans at a resolution of 4 cm^−1^. Baseline correction and Fourier self-deconvolution were performed using OMNIC™ 9 software (Thermo Fisher Scientific Inc., Waltham, MA, USA). For protein secondary structure, the Amide I (1700–1600 cm^−1^) region, was deconvoluted using Gaussian function with high sensitivity, applying a bandwidth of 22.5 and an enhancement factor of 3. The relative areas of individual peaks were determined using peak assignments by Singhi & Ozturk [32], with bands at 1600–1620 cm^−1^ attributed to side-chain vibrations, 1620–1645 cm^−1^ and 1685–1695 cm^−1^ to β-sheet structures, 1650–1660 cm^−1^ to α-helix structures, and 1660–1685 cm^−1^ to β-turn structures.

The starch fingerprint region (800–1200 cm^−1^) was similarly deconvoluted to evaluate short-range molecular organization of starch granules. Gaussian function with high sensitivity was applied using a bandwidth of 55.5 and an enhancement factor of 2.8 for all samples. The peak intensity ratios of R_995/1022_ and R_1047/1022_ were subsequently calculated.

#### 2.6.2. Fluorescence Intensity

Intrinsic fluorescence spectra were recorded for CPF dispersions following the method described by Singhi & Ozturk [32]. The CPF dispersions were diluted to a final concentration of 1 mg protein/mL using 0.01 M phosphate-buffered solution (pH 7.2). Fluorescence measurements were performed using a SpectraMax M5 microplate reader (Molecular Devices, San Jose, CA, USA) with excitation wavelength at 280 nm. Emission spectra were recorded over the range of 300–400 nm and the slit width was set to 5 nm.

### 2.7. Scanning Electron Microscopy

Lyophilized samples were affixed to non-conductive tape before coating with Au-Pd using a K575 ion sputter coater (Emitech, Montigny-le-Bretonneux, France). Scanning electron microscopy (SEM) was then performed using an Axia™ ChemiSEM™ Scanning Electron Microscope (Thermo Fisher Scientific, Waltham, MA, USA). The samples were processed at 2.00 kV accelerating voltage and 3.0 spot size. Micrographs were captured at 500× and 2000× magnifications.

### 2.8. Rheological Properties

Rheological characteristics of the samples were evaluated using a modified version of the method by Yurdagul & Ozturk [33], using an ARES-G2 rheometer (TA Instruments, New Castle, DE, USA) equipped with a DIN concentric cylinder geometry. All analyses were performed at a controlled temperature of 25 °C. The linear viscoelastic region (LVR) was initially determined through strain sweep measurement, in which shear strain varied from 0.01% to 100% at a constant angular frequency of 1 rad/s. Upon determining the LVR, frequency sweep test was conducted at a fixed shear strain of 0.1%, spanning a frequency range of 0.1–10 Hz, to characterize the viscoelastic response of the samples.

### 2.9. Thermal Properties

The thermal properties of lyophilized CPF samples were characterized using differential scanning calorimetry (DSC; Model Q2000, TA Instruments, USA). Samples (3–5 mg) were hermetically sealed in aluminum pans and an empty pan served as the reference. The samples were heated from 20 to 250 °C at 5 °C/min under nitrogen flow (20 mL/min). The transition midpoint temperature (T_m_) and transition enthalpy (ΔH) were determined from the DSC thermograms.

### 2.10. In Vitro Protein Digestibility

In vitro protein digestibility (IVPD) was assessed using a simulated gastric-intestinal digestion model according to the method reported by Singhi & Ozturk [32]. Microfluidized CPF dispersions were diluted to 2% (*w*/*v*) with deionized water and adjusted to pH 1.6 using 1 M HCl to simulate gastric conditions. Pepsin (≥2500 U/mg protein; Sigma-Aldrich, P6887) was added at 4% (*w*/*w*) relative to the protein content, and the mixture was incubated at 37 °C. Aliquots were collected at 0, 10, 30, 60, and 120 min to monitor gastric digestion. The pH was then gradually adjusted to 7.5 over approximately 2–3 min using 1 M NaOH to simulate intestinal conditions, followed by the addition of trypsin (≥10,000 U/mg protein; Sigma-Aldrich, T4799) at 4% (*w*/*w*) of the protein content. The samples were incubated at 37 °C and aliquots were collected at 150, 180, and 240 min. Enzymatic digestion was terminated by heating the samples at 95 °C for 15 min. Protein concentrations at each time point were quantified using the Bradford assay, and protein digestibility was calculated using the following equation.

(8) $$IVPD\left(\%\right)=\frac{{PC}_{0}-{PC}_{t}}{PC}\times 100\%$$

where *PC*_0_ and *PC_t_* represent the protein content at 0 and t min, respectively; *PC* represents the protein content of the initial dispersion. For subsequent principal component analysis, the IVPD value at 240 min (final digestion value) was used.

### 2.11. Statistical Analysis

Principal component analysis (PCA) was carried out using Prism 10 software (GraphPad, San Diego, CA, USA) based on physicochemical, functional, and digestibility parameters. The correlation among variables was visualized using a biplot.

All experiments were performed in triplicates using three separately microfluidized aliquots, unless stated otherwise. Statistical analyses were performed using Prism 10 software (GraphPad, CA, USA). Data were analyzed using one-way analysis of variance (ANOVA), with Tukey post hoc test at a significance level of *p* < 0.05.

## 3. Results and Discussion

### 3.1. Proximate Composition of CPF

The proximate composition of CPF is shown in Table 1. These values are consistent with those previously reported for chickpea flour [34], confirming the representative nature of the material used in this study. Minor deviations from previously reported values [35] may be attributed to differences in chickpea cultivar, growing conditions, and post-harvest processing parameters used during flour production, including milling and fractionation practices.

### 3.2. Physicochemical Properties of Microfluidized CPF

#### 3.2.1. Particle Size

**Figure 1:**
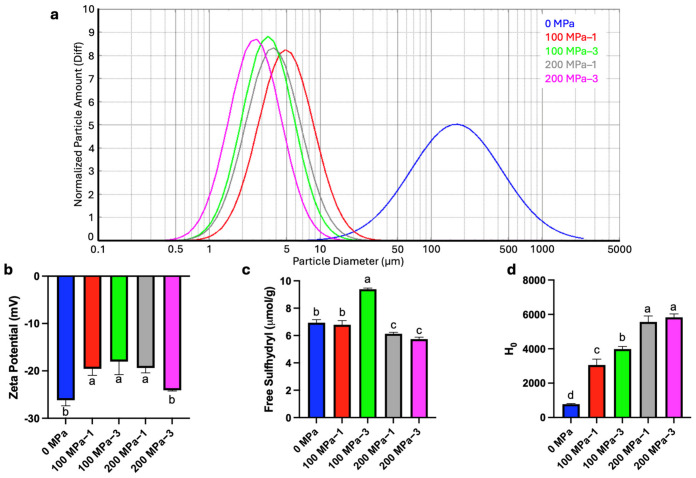
Effects of different microfluidization pressures and number of passes on physicochemical properties of 5% (*w*/*v*) chickpea flour dispersions in water: (**a**) particle size distribution, (**b**) zeta potential, (**c**) free sulfhydryl group content, and (**d**) surface hydrophobicity. Means with different letters indicate significant differences between treatments (*p* < 0.05). H_0_: Surface hydrophobicity. Error bars represent the standard deviation of means.

Microfluidization of CPF dispersions led to a dramatic decrease in particle size, regardless of applied pressure or number of passes (Figure 1a). This decrease can be attributed to the high shear forces and cavitation generated during microfluidization, which promote collisions that disperse large particles into finer fragments [28,36]. Increasing either processing pressure or the number of passes led to a further decrease in particle size, indicating a cumulative effect of energy input. Microfluidized samples exhibited markedly narrower particle size distributions compared to the control, reflecting enhanced dispersion uniformity. The results are in agreement with those on microfluidized legume flours. Adjei-Fremah et al. [21] observed smaller particle size and narrower distribution in whole cowpea flour microfluidized at 137 MPa for two passes. Similarly, Chen & Ozturk [28] reported a pressure-dependent decrease in particle size in partially defatted pumpkin seed flour after microfluidization. Collectively, these results confirm the effectiveness of microfluidization in disrupting complex flour matrices and improving dispersion homogeneity, which is critical for downstream functional performance.

#### 3.2.2. Zeta Potential

Zeta potential reflects the surface charge of dispersed particles and plays a key role in determining colloidal stability [37]. Microfluidization significantly altered the zeta potential of the CPF dispersions (*p* < 0.05), rendering the particles less negatively charged relative to the control, with the exception of 200 MPa–3 (Figure 1b). This shift toward less negative values is likely associated with microfluidization-induced structural disruption and resulting exposure of positively charged amino acid residues [38], such as arginine and lysine, which are present in a relatively high amount in chickpeas [7]. In contrast, the 200 MPa–3 sample exhibited a non-significant shift (*p* > 0.05) compared to the control, suggesting that the combination of high pressure and multiple passes led to protein aggregation, thereby masking charged residues and restoring a more negative surface potential. A similar pressure-dependent reversal in zeta potential was reported for perilla protein isolate, where aggregation occurred at pressures above 120 MPa [6]. In addition to protein structural changes, non-protein components in CPF, such as fiber and residual polysaccharides, may also contribute to the observed behavior after microfluidization [39]. While zeta potential is often linked to dispersion and emulsion stability [40], this trend was not observed in the microfluidized CPF (Section 3.3.2). This is likely due to the high viscosity of the continuous phase limiting flocculation, lowering any electrostatic stabilization effects due to the zeta potential. As such, a higher magnitude of zeta potential did not lead to greater emulsion stability. However, it is expected that zeta potential would play a greater role in dispersion and emulsion stability if the samples were diluted sufficiently.

#### 3.2.3. Free Sulfhydryl Groups

The content of free sulfhydryl groups experienced a significant increase (*p* < 0.05) only in the 100 MPa–3 sample, while no significant increases were observed for the other samples (Figure 1c). This could be attributed to both protein unfolding and disulfide bond cleavage under moderate pressure and repeated passes, exposing previously buried sulfhydryl groups [41]. In contrast, microfluidization at 100 MPa for a single pass likely provided insufficient energy to induce substantial disulfide bond disruption, leading to no significant change (*p* > 0.05) in free sulfhydryl group content. On the other hand, microfluidization at 200 MPa likely promoted disulfide bond formation due to the high pressure, leading to a lower free sulfhydryl group content (*p* < 0.05). These results indicate that microfluidization at lower pressures for multiple passes is more effective for increasing free sulfhydryl group content. Melchior et al. [42] similarly observed that moderate-pressure homogenization applied over multiple cycles maximized free sulfhydryl exposure in pea protein concentrates. Increased sulfhydryl content is particularly advantageous for applications, such as extrusion-based meat analogues, where thiol-mediated crosslinking contributes to fibrous structure formation and textural development [43].

#### 3.2.4. Surface Hydrophobicity

Surface hydrophobicity (H_0_) increased significantly with microfluidization in a pressure- and pass-dependent manner (Figure 1d). The control sample showed the lowest values, whereas 200 MPa–3 exhibited the highest, corresponding to an ~7.8-fold increase. Notably, substantial increases were also observed at 100 MPa, particularly for 100 MPa–3, indicating that even moderate pressure combined with multiple passes is sufficient to expose previously buried non-polar hydrophobic residues. The progression from the control to 100 MPa–3 and 200 MPa–3 highlights the synergistic effects of pressure intensity and pass number. The similarity in H_0_ values between 200 MPa–1 and 200 MPa–3 (*p* > 0.05) suggests the presence of a pressure-threshold, beyond which additional passes yield diminishing returns. Once this pressure threshold is reached, additional cycling may instead promote partial re-aggregation of unfolded protein structures, consistent with unfolding–aggregation behavior reported in legume proteins treated with high-pressure homogenization [44,45]. Wang et al. [46] also demonstrated that high-pressure homogenization dissociates and rearranges large insoluble protein aggregates and increases hydrophobic exposure through disruption of weak non-covalent interactions (e.g., hydrogen bonding, electrostatic forces), enabling hydrophobic side chains to migrate toward the protein-solvent interface [44]. The elevated hydrophobicity at 200 MPa correlates with enhanced intermolecular protein–protein interactions through hydrophobic aggregation, which are closely linked to improved gel strength and viscoelastic behavior in legume systems treated with high-pressure homogenization [47]. Collectively, these findings indicate that high-pressure processing promotes progressive protein unfolding and structural rearrangement in CPF, resulting in substantial exposure of hydrophobic groups with direct implications for functionality in structured plant-based food applications.

### 3.3. Functional Properties of Microfluidized CPF

#### 3.3.1. Protein Solubility

**Figure 2:**
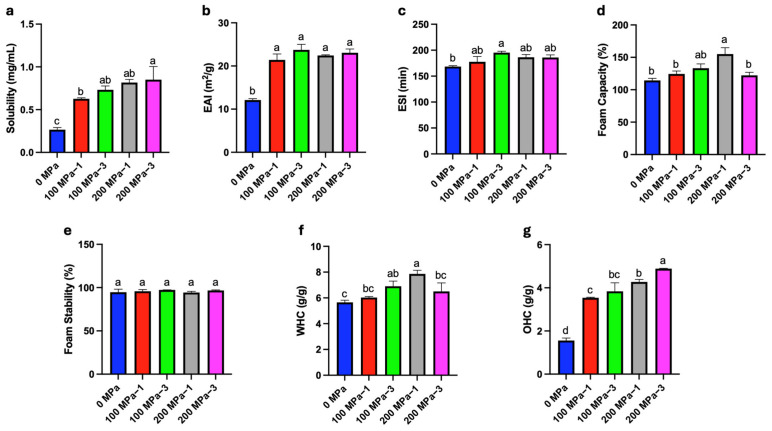
Effects of microfluidization pressure and number of passes on functional properties of chickpea flour. (**a**) Protein Solubility, (**b**) Emulsion Activity Index, (**c**) Emulsion Stability Index, (**d**) Foam Capacity, (**e**) Foam Stability, (**f**) Water-Holding Capacity, and (**g**) Oil-Holding Capacity. Means with different letters indicate significant differences between treatments (*p* < 0.05). Error bars represent the standard deviation of means.

The solubility of CPF was significantly enhanced with microfluidization (Figure 2a). The control exhibited the lowest solubility (0.27 mg/mL), whereas all treated samples showed progressive increases with both pressure and cycle number. The enhancement in solubility can be primarily attributed to the physical disruption of large protein aggregates and particle size reduction during microfluidization through the combined effects of intense shear, turbulence, and cavitation [48], which increased surface area available for protein-water interactions and improved hydration capacity, thereby enhancing solubility. Similar phenomena have been reported for chickpea and other plant-based proteins treated with high-pressure homogenization, where exposure previously buried amino acid residues and reactive groups promotes hydration, dispersion, and solubility [45]. This substantial increase in protein solubility observed in microfluidized CPF suggests potential utility for improving protein extraction efficiency and broadening its applicability in aqueous food systems.

#### 3.3.2. Emulsifying Properties

The emulsifying activity index (EAI) and emulsion stability index (ESI) of the CPF dispersions are presented in Figure 2b,c. The control exhibited the lowest EAI (12.11 m^2^/g), whereas all treated samples displayed approximately 2-fold higher values, reflecting improved interfacial activity following processing. This enhancement is likely associated with particle fragmentation and greater exposure of hydrophobic groups, which facilitate particle migration and absorption at the oil-water interface [49,50]. However, no statistically significant differences in EAI were detected among the microfluidized samples (*p* > 0.05), suggesting that varying processing conditions did not differentially influence the proteins’ ability to form emulsions. Similar observations were reported for pea protein isolates treated by high-pressure homogenization, where increasing the treatment intensity (60–100 MPa) markedly enhanced solubility and water/oil holding capacity but only weakly affected interfacial tension and emulsifying capacity [51].

Microfluidization moderately improved emulsion stability, although the effects were treatment-dependent. The 100 MPa–3 sample exhibited significantly higher ESI than the control, whereas other treated samples showed intermediate values without clear separation. This trend is likely influenced by differences in viscosity and network strength (discussed with rheology data in Section 3.6). An increase in viscosity, caused by improved protein and carbohydrate hydration, can slow flocculation. Stronger networks, formed by interactions between both proteins and carbohydrates, can also suppress droplet movement and flocculation in accordance with Stokes’ Law [52]. In contrast, the limited improvement in ESI for the 200 MPa-3 samples may be attributed to particle aggregation effects, which can hinder droplet size reduction and compromise emulsion stability despite increased viscosity.

#### 3.3.3. Foaming Properties

Foaming capacity (FC) of the CPF increased notably after microfluidization (Figure 2d). The control showed an FC of 114%, while all microfluidized samples exhibited higher values, indicating improved air incorporation. Treatments at 100 MPa resulted in moderate increases (124–133%), whereas the highest FC (155%) was observed for the 200 MPa–1 sample. These improvements are attributed to pressure-induced structural modifications that enhance interfacial activity and facilitate more rapid film formation around air bubbles, consistent with previous work on legume proteins treated with high-pressure homogenization [53]. However, FC decreased (122%) for the 200 MPa–3 sample, suggesting that excessive pressure and repeated passes may induce partial reaggregation of protein and carbohydrate structures, thereby limiting the ability of the system to efficiently incorporate and potentially stabilize air during whipping. Similar pressure-dependent declines in foaming performance have been reported for oat proteins subjected to excessively high microfluidization pressures [54].

Foam stability (FS) remained consistently high across all samples (94–97%), with no statistically significant differences (*p* > 0.05) among treatments (Figure 2e). This indicates that microfluidization enhanced foam formation without compromising foam stability. Comparable behavior was observed for chickpea-derived systems treated with high-pressure homogenization, including aquafaba, which inherently form strong interfacial networks consisting of both proteins and carbohydrates, and are capable of resisting foam collapse [55]. Overall, microfluidization effectively improved foam ability while preserving structural stability.

#### 3.3.4. Water- and Oil-Holding Capacities

WHC is a key determinant of texture, juiciness, and storage stability in food systems. It is primarily determined by the availability of hydrophilic binding sites and the structural characteristics of the food matrix [21]. As shown in Figure 2f, the WHC of CPF increased significantly (*p* < 0.05) with increasing microfluidization pressure and number of passes. This enhancement is attributed to microfluidization-induced disruption of the polysaccharide matrix, reduced particle size, promoted micropore formation and surface area, and increased exposure of carboxyl and hydrated hydroxyl groups [56], all of which likely increased the number of water-binding sites available and promoted protein-water and carbohydrate-water interactions [57]. In addition, the WHC measurement conditions (60 °C for 40 min) may have contributed to the observed increase through partial starch gelatinization and protein denaturation, which can further enhance water retention capacity. Similar improvements in WHC following high-pressure microfluidization have been previously reported for wheat bran [58] and cowpea flour [21]. However, further intensification of treatment (200 MPa–3) resulted in a slight but significant decrease in WHC (*p* < 0.05), suggesting that excessive structural disruption may reduce water binding affinity, limiting water retention, as reported for other high-pressure-treated plant materials [6].

OHC is also an important functional property, mostly of polysaccharides, and is governed by their chemical structure and physicochemical attributes, including surface morphology and hydrophobicity [54]. Microfluidization significantly increased the OHC of CPF (Figure 2g), indicating an enhanced ability of the polysaccharide matrix to retain oil through physical entrapment and capillary interactions. These results demonstrate that processing pressure and number of passes were critical factors influencing this behavior. OHC exhibited a positive linear relationship with both pressure and number of passes. This enhancement can be associated with increased exposure of hydrophobic regions capable of binding with lipid molecules and enhanced surface roughness, which facilitate lipid absorption [59]. These findings are in line with the surface hydrophobicity trends (Figure 1d) and aligns with a previous report on microfluidized cowpea flour [21]. Overall, these results highlight the ability of microfluidization to selectively enhance hydration and fat-binding properties of chickpea flour for plant-based food applications.

### 3.4. Molecular Structure

**Figure 3:**
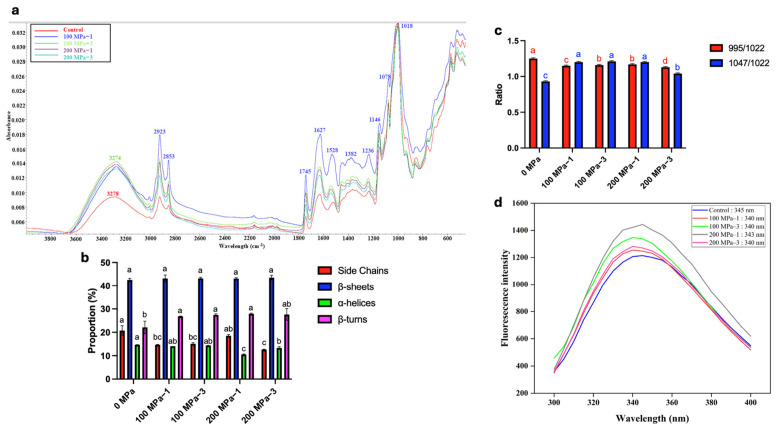
Effects of different microfluidization pressures and number of passes on structural characteristics of lyophilized chickpea flour: (**a**) Fourier-transform infrared spectra, (**b**) proportions of different protein secondary structures, (**c**) peak absorption ratios at wavenumbers of 995/1022 and 1047/1022, (**d**) intrinsic fluorescence spectra with maximum emission wavelengths (λ_max_) of each sample. Means with different letters indicate significant differences (*p* < 0.05) between treatments, within the same type of protein secondary structure or peak absorption ratio. Error bars represent the standard deviation of means.

As presented in Figure 3a, the FTIR spectra of the control and microfluidized CPF samples were largely comparable, indicating that microfluidization did not induce significant changes in the overall chemical structure of CPF. The broad absorption band in the 3000–3500 cm^−1^ region corresponds to amide A and amide B vibrations, primarily associated with N–H stretching [60]. Bands observed in the 3000–2840 cm^−1^ region are attributed to C–H stretching vibrations [61]. The prominent absorption band in the 1700–1600 cm^−1^ region, corresponding to the amide I band, arises mainly from C=O stretching vibrations of the protein backbone [62]. The amide II band peaks, associated with N–H bending and C–N stretching vibrations, were located between 1532 and 1525 cm^−1^. All samples exhibited a characteristic peak at approximately 1382 cm^−1^, which may be attributed to C–H bending vibrations within the 1300–1500 cm^−1^ region [63]. In the carbohydrate fingerprint region (1200–800 cm^−1^), a prominent peak at approximately 1018 cm^−1^ was consistently observed across all samples, corresponding to C–O stretching vibrations of glycosidic linkages [64]. Additional characteristic bands related to C–C and C–O–C stretching of glycosidic bonds at approximately 1146 cm^−1^, as well as C–O–H in-plane bending at 1078 cm^−1^, were also identified [65].

#### 3.4.1. Protein Secondary Structure

The secondary structure is primarily stabilized by intermolecular hydrogen bonds, which can be broken by the intense shear, turbulence, and cavitation forces generated during microfluidization, potentially leading to the conversion between different secondary structure conformations [66]. Deconvolution of the Amide I band (1700–1600 cm^−1^) revealed that the untreated CPF predominantly consisted of β-sheet structures (43%), with lower proportions of α-helices (15%), β-turns (22%), and side chains (21%) (Figure 3b), which are comparable to the previous reported values [24]. Microfluidized CPF showed a significant decrease (*p* < 0.05) in side chains and α-helices, while no significant change (*p* > 0.05) was observed in β-sheet content. On the other hand, the proportions of β-turns increased significantly (*p* < 0.05) following treatment. The decrease in α-helix content is commonly associated with increased flexibility, as α-helices represent relatively rigid structural elements [67]. Enhanced protein flexibility facilitates structural adaptation at interfaces and has been linked to improved emulsifying performance in plant-based protein systems [68], affecting CPF’s emulsion capacity (Figure 2b). This increase in structural flexibility is further corroborated by the enhanced solubility, WHC, and OHC in treated samples (Figure 2a,f,g), indicating greater exposure of both hydrophilic and hydrophobic residues previously buried within the protein core. Furthermore, the significant increase in β-turn content in treated samples indicates protein unfolding induced by microfluidization, which is often associated with the exposure of hydrophobic regions [69]. This interpretation is corroborated by the surface hydrophobicity results (Figure 1d), which exhibited a clear increasing trend with increasing pressure and number of passes.

#### 3.4.2. Starch Short-Range Ordered Structure

The FTIR spectral region between 1200 and 800 cm^−1^ corresponds to the starch fingerprint region and is used to evaluate short-range molecular order in starch-based systems. In this region, the absorption bands at 1047 and 1022 cm^−1^ are predominantly associated with the crystalline and amorphous domains of starch granules, respectively, whereas the band at 995 cm^−1^ is related to the hydration-sensitive double-helical conformations of starch chains [70]. Consequently, the absorbance ratio R_1047/1022_ is used to assess the degree of short-range crystalline order, while R_995/1022_ reflects the conformational stability and organization of double-helical structures within the crystalline lamellae [71]. As shown in Figure 3c, microfluidization significantly (*p* < 0.05) altered the short-range ordered structure of the starch fraction present in CPF. Specifically, all treated samples exhibited an increase in the R_1047/1022_ ratio, with the greatest increase (30.1%) observed in samples treated at 100 MPa for three passes. This increase suggests that microfluidization promoted the formation of more ordered crystalline structure within the starch granules. Similar observations have been reported in rice starch systems, where microfluidization treatment at 100 MPa promoted the formation of more short-range ordered starch structures [72]. In contrast, the R_995/1022_ ratio decreased in all treated samples, reaching a maximum reduction of 9.6% at 200 MPa after three passes. This reduction indicates partial disruption and reduced stability of starch double-helical conformations following microfluidization treatment. The stronger gel-like behavior observed in the rheological measurements (Section 3.6) further supports these findings, suggesting that the increased short-range ordered structure contributed to improved network strength and structural stability in the microfluidized CPF dispersions. These results demonstrate that microfluidization modified the molecular organization of the starch fraction in CPF, affecting both the short-range ordered structure and the stability of double-helical arrangements within the crystalline regions.

#### 3.4.3. Protein Tertiary Structure

Intrinsic fluorescence spectroscopy was employed to evaluate changes in the tertiary structure of CPF. Fluorescence emission is mainly governed by aromatic amino acids (i.e., tryptophan (Trp), tyrosine (Tyr), and phenylalanine (Phe)), and changes in fluorescence intensity reflect conformational alterations related to alterations in the polarity of environment surrounding these residues [73]. As shown in Figure 3d, microfluidization caused a pronounced and progressive increase in the maximum fluorescence intensity of CPF compared to the control, with the effect strengthening as both processing pressure and number of passes increased. This increase in the intensity indicates partial unfolding of protein molecules under the intense mechanical forces generated during microfluidization, leading to disruption of intramolecular hydrophobic interactions and increased exposure of previously buried aromatic residues to the aqueous environment [74]. In addition, the increased fluorescence intensity may also be partially attributed to the dissociation of protein aggregates into smaller structural units, as evidenced by the reduced particle size (Figure 1a). Aggregate disruption increases the number of fluorescent chromophores contributing to the overall signal [75]. However, at the most intensive processing condition (200 MPa–3), a decline in fluorescence intensity was observed, which may be attributed to fluorescence quenching effects caused by excessive unfolding or reaggregation, as previously reported by Fan et al. [76] and Liu et al. [77]. Shifts in the maximum emission wavelength (λ_max_) further supported these structural changes. The control CPF showed a λ_max_ at 345 nm, whereas all treated samples exhibited a decrease in λ_max_ irrespective of pressure and number of passes. Since λ_max_ values above 330 nm generally indicate that chromophoric groups (particularly Trp residues) are in a relatively polar environment, the observed blue shift in λmax after the microfluidization treatment suggests changes in the tertiary structure that positioned aromatic residues within a comparatively less polar microenvironment [78]. Overall, the pressure- and pass-dependent fluorescence behavior provides clear evidence that microfluidization induces cumulative modifications in the tertiary structure of CPF proteins, involving both molecular unfolding and aggregate disruption. These structural changes underpin the observed improvements in functional properties and highlight the effectiveness of microfluidization as a physical modification strategy for enhancing the performance of plant-based protein ingredients.

### 3.5. Surface Morphology

**Figure 4:**
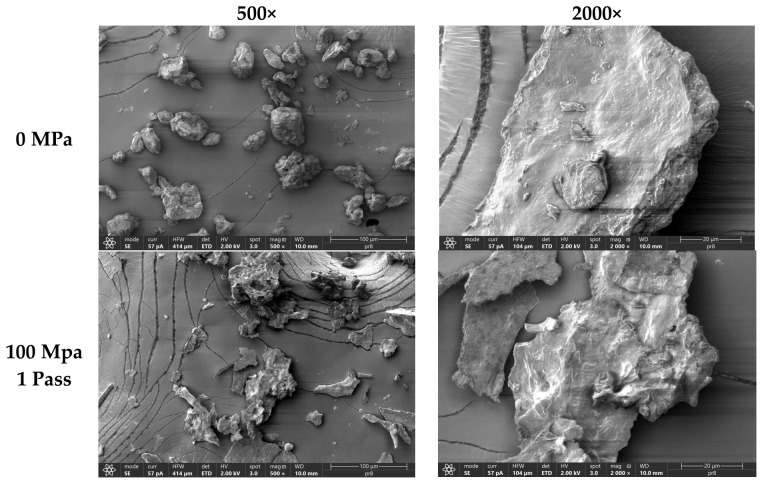

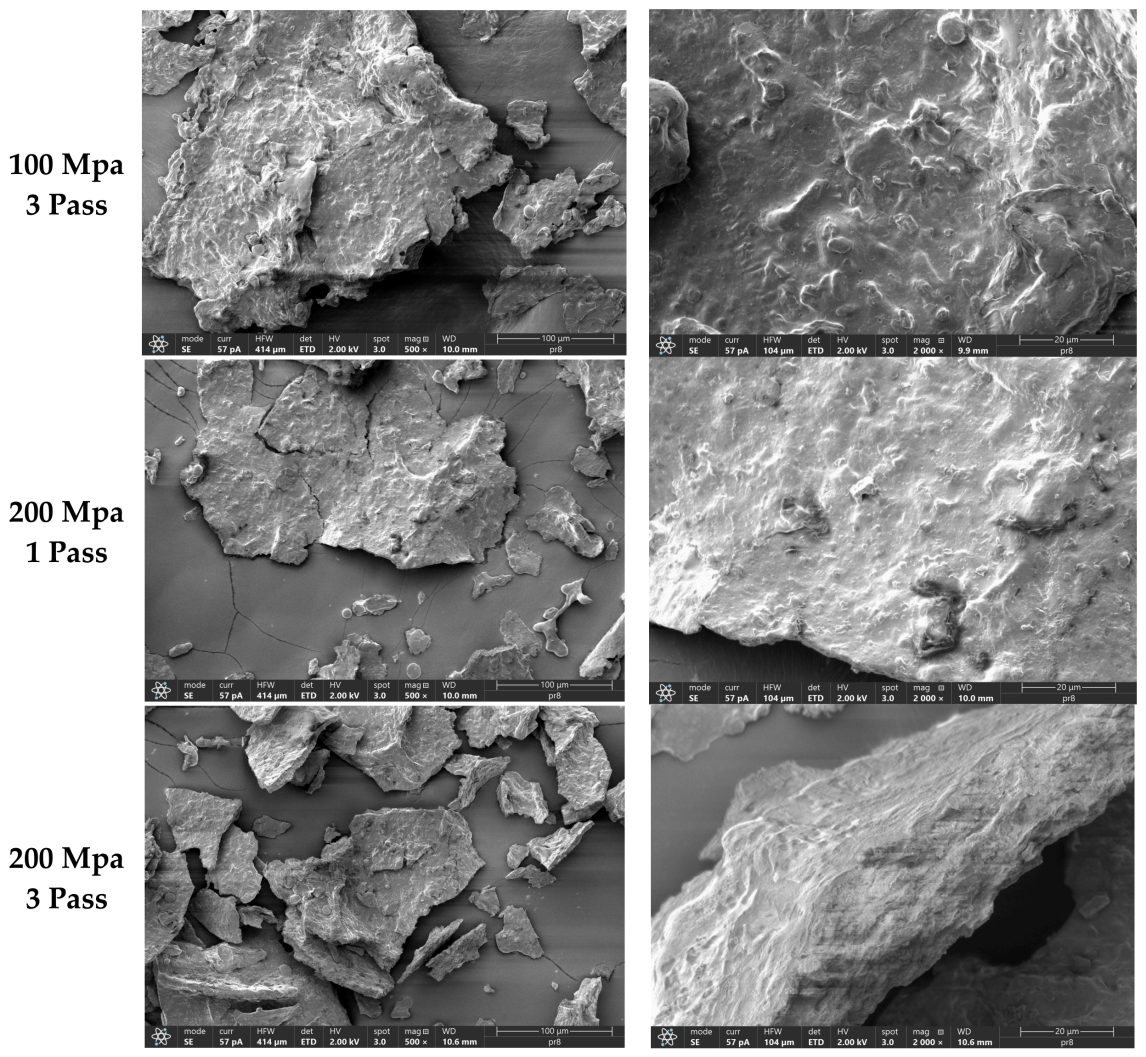
Scanning electron micrographs of lyophilized chickpea flour at 500× (**left**) and 2000× (**right**) magnification levels after microfluidization at different pressures and number of passes.

SEM micrographs showing the surface morphology of lyophilized CPF are presented in Figure 4. Microfluidization markedly altered the microstructure of CPF, producing particles with a more irregular, fragmented, and rough surface compared to the relatively smooth morphology of the untreated sample. These morphological changes could be attributed to intense pressure, shear, turbulence, and cavitation forces generated during the microfluidization, which promote particle fragmentation and surface erosion. The increased surface roughness observed in microfluidized samples probably resulted in a greater surface area to volume ratio, thereby improving interactions between CPF and either oil or water. This structural effect is consistent with the observed improvements in protein solubility, WHC, and OHC (Figure 2a,f,g). Additionally, the expanded surface area likely exposed previously buried hydrophobic regions, thereby contributing to the increased surface hydrophobicity (Figure 1d) [78]. The sample treated at the most intensive condition (200 MPa–3) exhibited a comparatively smoother and less porous surface, suggesting the occurrence of pressure-induced reaggregation. Similar observations were reported for almond proteins, where microfluidization led to a more irregular and porous texture at moderate pressures, but these effects diminished at high pressures (>120MPa) due to particle reaggregation [41]. Likewise, disruption of smooth, compact surfaces of insoluble dietary fiber was also noted by Chen et al. [79] after microfluidization.

### 3.6. Rheological Properties

**Figure 5:**
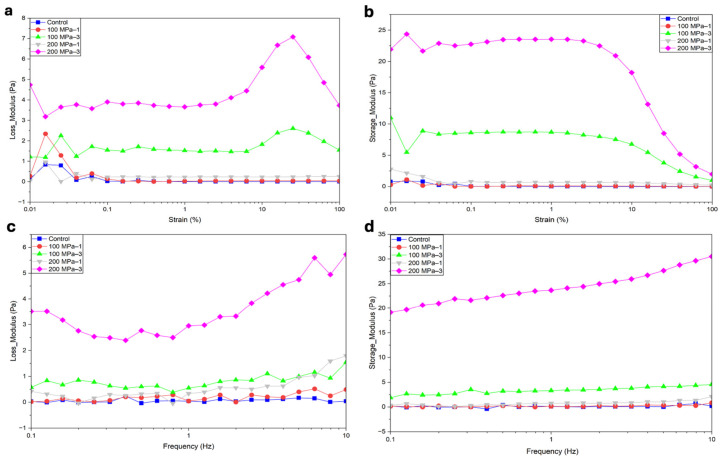
Effects of different microfluidization pressures and number of passes on rheological characteristics of chickpea flour dispersed in water (5%, *w*/*v*): (**a**,**b**) amplitude sweep measurements, and (**c**,**d**) frequency sweep measurements.

Oscillatory amplitude sweeps were performed to determine the linear viscoelastic region (LVR) of the CPF dispersions (Figure 5a,b). For all samples, the storage (G′) and loss (G″) moduli displayed a plateau at low strain amplitudes, indicating structurally stable networks resistant to small deformations. Based on these observation, a strain amplitude of 0.1% was selected for subsequent frequency sweep tests to ensure measurements within the LVR and reflecting only the intrinsic viscoelastic properties of the systems, consistent with previous reports on defatted chickpea flour-based gels [80].

Frequency sweep measurements (Figure 5c,d) revealed G′ values that remained consistently higher than G″ over the entire angular frequency range (0.1–10 Hz) for all samples, indicating predominantly weak gel-like behavior. The dominance of elastic (G′) over viscous (G″) response indicates the presence of interconnected three-dimensional networks capable of storing mechanical energy and withstand deformation, in agreement with observations for other pulse protein systems [81]. Distinct differences in network strength were observed among the treatments. The 200 MPa–3 sample exhibited the highest G′ and G″ values across the entire frequency range, indicating the formation of a substantially stronger and more elastic gel network, with limited molecular relaxation.

This strengthening is attributed to the combined effect of high pressure and repeated passes, which promote protein unfolding, exposure of hydrophobic regions, and enhanced intermolecular aggregation, collectively increasing interactions between water, proteins, and carbohydrates. Microfluidization led to a decreased particle size, which would have increased interactions between CPF and water, leading to a stronger gel network. Additionally, partial starch gelatinization may have further contributed to network reinforcement [20]. Similar results have been reported for chickpea protein dispersions and aquafaba systems subjected to high-pressure homogenization [82,83]. In contrast, the control and samples treated at lower pressure or fewer passes exhibited substantially lower moduli and weaker frequency dependence, indicative of loosely associated gel-like structures with limited structural connectivity. Among these, 100 MPa–3 showed modestly higher moduli values, indicating partial network development insufficient to match the structural integrity achieved under the most intensive treatment. This weaker gel behavior may be attributed to insufficient particle disruption and limited formation of continuous structural networks under milder processing conditions, leading to larger residual particle size and reduced intermolecular interactions. This behavior may also be related to limited starch gelatinization under milder processing conditions, as previously reported in starch-based systems where lower-intensity microfluidization resulted in reduced structural disruption and a lower degree of gelatinization, thereby weakening pasting and rheological properties [84].

Overall, these results demonstrate that microfluidization at high pressure combined with extended treatment duration is critical for maximizing viscoelastic network strength in chickpea-based dispersions, showing its potential for tailoring texture in plant-based food applications.

### 3.7. Thermal Properties

**Figure 6:**
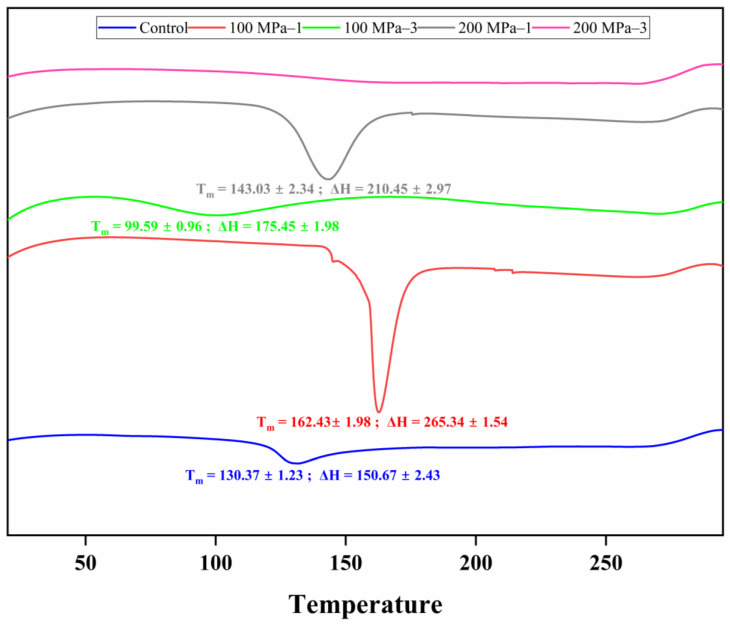
Effects of microfluidization pressure and number of passes on differential scanning calorimetry thermographs of chickpea flour. T_m_: transition midpoint temperature (°C); ΔH: transition enthalpy (J/g).

The thermal properties of CPF, namely the transition midpoint temperature (T_m_) and transition enthalpy (ΔH), were significantly (*p* < 0.05) affected by the microfluidization treatment. T_m_ indicates the thermodynamic stability of the carbohydrate-protein matrix, with higher values reflecting greater resistance to thermal unfolding [85]. Single-pass microfluidization enhanced the thermal stability of CPF compared to the control (Figure 6), with 100 MPa treatment leading to the maximum T_m_ value (162.43 °C). This suggests that moderate pressure treatment may promote favorable structural rearrangements, such as enhanced molecular packing or strengthened intermolecular interactions, resulting in a more thermally stable conformation [73]. However, further increasing the pressure to 200 MPa resulted in a reduction in T_m_ (143.03 °C), although the value remained higher than that of the control (130.37 °C), indicating the onset of pressure-induced structural destabilization. Interestingly, the T_m_ for the untreated CPF in this study was considerably higher than values reported for chickpea protein isolates (98–99 °C) [86]. This difference is likely due to the presence of other non-protein components, such as starch and fiber, which can restrict protein mobility and confer additional thermal protection within whole-flour matrices [87]. Moreover, increasing the number of microfluidization passes exhibited a clear negative effect on thermal stability, highlighting the cumulative disruptive impact of repeated mechanical and shear forces on CPF structure. The reduced stability can be attributed to the formation of unfolded or partially unfolded protein-carbohydrate conformations, which are inherently more susceptible to thermal denaturation [88]. Notably, CPF treated at 200 MPa for three passes did not exhibit a distinct endothermic transition within the investigated temperature range, suggesting extensive or complete denaturation of the major protein and carbohydrate fractions.

The ΔH, which represents the energy required to disrupt ordered molecular structures during heating, was significantly higher (*p* < 0.05) in all treated samples compared to the control. The highest ΔH value (265.32 J/g) was observed for CPF treated at 100 MPa for one pass, indicating the formation of a more ordered and thermally stable structure. This finding is consistent with the enhanced short-range molecular order observed from FTIR analysis (Figure 3c). The increase in ΔH may also be associated with stronger carbohydrate-protein interactions involving exposed hydrophobic and hydrophilic groups (Figure 1d and Figure 2a), thereby increasing the energy required to disrupt crystalline and ordered regions during heating. In contrast, increasing processing intensity reduced both ΔH and T_m_, confirming that excessive microfluidization disrupts structural organization and thermal stability. Due to the absence of a clear endothermic peak, ΔH could not be determined for CPF treated at 200 MPa for three passes. Overall, the thermal behavior of CPF was found to be strongly dependent on microfluidization pressure intensity and processing intensity. Moderate microfluidization conditions promoted structural rearrangements that enhanced thermal stability, whereas excessive pressure and repeated passes disrupted the organized carbohydrate–protein matrix, resulting in reduced thermal resistance Therefore, optimization of treatment conditions is critical to balance structural modification and thermal stability.

### 3.8. In Vitro Protein Digestibility

**Figure 7:**
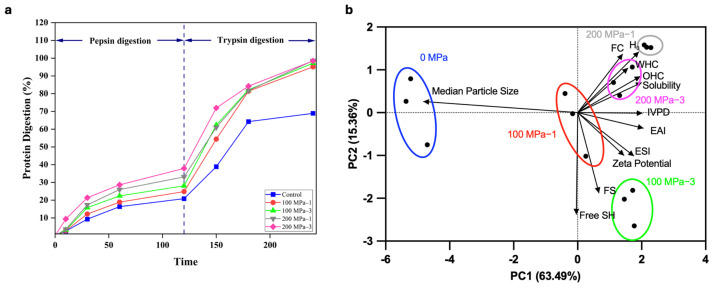
(**a**) Effects of microfluidization pressure and number of passes on in vitro protein digestibility of chickpea flour. (**b**) Principal component analysis (PCA) biplot of physicochemical and functional properties of microfluidized chickpea flour. Free SH: Free Sulfhydryl Groups; H_0_: Surface Hydrophobicity; EAI: Emulsion Activity Index; ESI: Emulsion Stability Index; FC: Foam Capacity; FS: Foam Stability; WHC: Water-Holding Capacity; OHC: Oil-Holding Capacity; IVPD: In Vitro Protein Digestibility.

Microfluidization significantly enhanced (*p* < 0.05) the IVPD of chickpea protein during both the simulated gastric and intestinal digestion phases even though the protein portion was embedded in the whole flour with other carbohydrate structures (Figure 7a). The effect was particularly pronounced during the intestinal phase, where microfluidized samples reached approximately 98% digestibility, compared to 67% for the control, demonstrating the substantial impact of microfluidization on protein bioaccessibility. During the gastric phase (0–120 min), IVPD increased progressively with both processing pressure and number of passes, indicating that intensified microfluidization rendered chickpea proteins more susceptible to pepsin-mediated hydrolysis. A similar trend was observed during the early intestinal phase (120–180 min). However, in the later stages of intestinal stage (180–240 min), no significant differences (*p* > 0.05) were detected among samples treated at higher pressures or with additional passes, suggesting that enzymatic hydrolysis approached saturation as readily accessible cleavage sites became depleted [89]. The enhanced IVPD of microfluidized chickpea protein can be attributed to multiple structural modifications. The marked reduction in particle size (Figure 1a) and loosening of the protein microstructure (Figure 4) increased enzyme-substrate interactions, facilitating proteolytic action during digestion [41]. In addition, the treatment induced exposure of previously buried hydrophobic amino acid residues, as confirmed by the increased OHC (Figure 2g) and surface hydrophobicity (Figure 1d). These exposed residues likely serve as preferred cleavage sites for proteolytic enzymes such as pepsin and trypsin, further enhancing hydrolysis [90].

### 3.9. Principal Component Analysis

To elucidate multivariable correlations among the physicochemical and functional characteristics of microfluidized CPF, principal component analysis (PCA) was conducted (Figure 7b). The first two principal components accounted for 78.85% of the total variance, with PC1 accounting for 63.49% and PC2 for 15.36%. Particle size generally showed a strong negative correlation with most functional properties, particularly IVPD and EAI. This relationship reflects the role of particle size reduction in increasing surface are, which facilitates more extensive interactions between protein and water, oil, or air, thereby leading to improvements in solubility, EAI, FC, WHC, and OHC [54]. The decreased particle size, coupled with improved solubility and increased surface hydrophobicity, further facilitated protein migration to the oil-water or air-water interfaces, improving EAI and FC [91]. Additionally, the expanded surface area allowed for greater enzyme-substrate binding, leading to improved IVPD [41]. Overall, microfluidized samples clustered in the positive direction of PC1, corresponding to broad improvements in functional properties. Differences in microfluidization pressure and number of passes primarily affected sample distribution along PC2, indicating condition-specific effects. 100 MPa–3 treatment led to a more negative PC2 score, associated with better FS and higher free sulfhydryl group content, indicative of enhanced protein reactivity and network-forming potential. On the other hand, 200 MPa–1 treatment showed a more positive PC2 score, correlating with improved solubility, FC, WHC, and OHC. These results show the importance of tailoring the microfluidization parameters to target specific functional outcomes. For instance, lower pressures combined with multiple passes would be advantageous for applications such as meat analogs that benefit from higher free sulfhydryl group content, while moderate to high pressures may be preferable when maximizing water and oil retention to enhance juiciness.

## 4. Conclusions and Future Directions

This study examined the influence of microfluidization pressure and number of passes on the physicochemical and functional characteristics of CPF. Microfluidization effectively reduced particle size and induced protein unfolding, leading to the formation of more irregular and porous surface structures. These structural modifications increased surface hydrophobicity and enhanced the amphiphilic nature of the proteins, thereby improving their emulsifying and foaming capacities. The elevated surface hydrophobicity also led to a significant enhancement in OHC (up to 210%), reflecting stronger interactions with nonpolar solvents. In addition, these changes promoted greater protein-water interactions, yielding substantial increases in protein solubility (210%) and WHC (40%). Increased surface area further enhanced IVPD of CPF (about 45%) and contributed to formation of a stronger network. Overall, these results demonstrate that microfluidization is an effective non-thermal strategy for enhancing techno-functional properties of whole chickpea flour, supporting its applications in plant-based food formulations. The findings reported here are representative of the effects of microfluidization on 5% (*w*/*v*) CPF dispersions; however, variations in concentration of CPF may yield processing outcomes. Future studies should therefore explore concentration-dependent effects and assess performance in complex food matrices to further optimize microfluidization conditions for targeted functional enhancements.

## Figures and Tables

**Table 1:** Proximate composition of chickpea flour.

Component	Proximate Composition (%)
Moisture	10.21 ± 0.02
Lipids	5.80 ± 0.06
Proteins	20.05 ± 0.07
Ash	2.81 ± 0.03
Carbohydrates	61.16 ± 0.05

Values are presented as mean ± standard deviation (SD).

## Data Availability

The original contributions presented in this study are included in the article. Further inquiries can be directed to the corresponding author.
